# Biomechanical effects of screw loosening after lumbar PEEK rod and titanium rod fixation: a finite element analysis

**DOI:** 10.3389/fbioe.2025.1533088

**Published:** 2025-03-17

**Authors:** Guozheng Jiang, Shuyang Wang, Luchun Xu, Zeyu Li, Ningning Feng, Ziye Qiu, Yongdong Yang, Xing Yu

**Affiliations:** Dongzhimen Hospital, Beijing University of Chinese Medicine, Beijing, China

**Keywords:** pedicle screw loosening, finite element, biomechanics, PEEK rods, titanium rods

## Abstract

**Objective:**

Screw loosening is a common complication following lumbar spine fixation surgery, yet the biomechanical outcomes after screw loosening remain rarely reported. This study aims to utilize finite element (FE) models to compare the biomechanical performance of PEEK rod dynamic fixation and titanium rod rigid fixation in the postoperative lumbar spine, exploring potential biomechanical mechanisms for re-stabilization of loosened screws.

**Methods:**

A FE model of the lumbar spine from L3 to the sacrum was developed using CT image segmentation. Four L4-S1 fixation models were constructed: PEEK rod dynamic fixation (PEEK model), titanium rod rigid fixation (titanium model), PEEK rod with pedicle screw loosening (PEEK-PSL model), and titanium rod with pedicle screw loosening (titanium -PSL model). A preload of 300 N was applied to the superior surface of L3. Stress distributions in the intervertebral discs, facet joints, pedicle screws, and rods were calculated to evaluate the biomechanical effects of different fixation methods.

**Results:**

Across four physiological loading conditions, the stress differences in intervertebral discs, facet joints, and nucleus pulposus between the PEEK model and titanium model were minimal. However, vertebral body stress was significantly higher in the PEEK model, whereas screw and rod stresses were greater in the titanium model. Screw loosening further increased stress in all models. The S1 screw in the PEEK-PSL model exhibited lower and more uniform stress, while stress was concentrated at the screw-rod junction in the titanium-PSL model.

**Conclusion:**

The PEEK rod fixation system demonstrated superior stress distribution, reducing stress concentration risks and improving stability while minimizing screw loosening rates. In contrast, the titanium rod system offers advantages in scenarios requiring high rigidity, potentially making it more suitable for patients with greater stability needs.

## 1 Introduction

Lumbar fusion surgery is one of the classic surgical methods for treating lumbar degenerative diseases. It effectively alleviates pain, restores spinal stability, and demonstrates favorable short- and mid-term clinical outcomes (Kwon et al., 2022; Chan et al., 2023). To date, lumbar fusion remains the “gold standard” for managing lumbar degenerative conditions and is widely performed worldwide (Fenton-White, 2021). By stabilizing the unstable segments through fusion, pathological displacement between lumbar structures can be prevented, maintaining spinal stability and reducing nerve compression and localized pain (de Kunder et al., 2018).

However, complications such as adjacent segment degeneration (ASD) have increasingly drawn attention during long-term follow-up (Shin et al., 2024). ASD refers to degenerative changes in the non-fused adjacent segments caused by additional mechanical stress following fusion surgery (Hilibrand and Robbins, 2004; Lau et al., 2021). Lumbar fusion alters the normal stress distribution and transmission of the lumbar spine, leading to increased stress on adjacent segments. This accelerates the degeneration of intervertebral discs, vertebral endplate cartilage, and facet joints, potentially necessitating revision surgery (Yu et al., 2024a).

In recent years, non-fusion dynamic fixation techniques have been introduced into clinical practice. These techniques aim to maintain the stability of the fixed segment while preserving partial physiological motion of the spine, thereby reducing the load on adjacent segments and lowering the incidence of ASD (Li et al., 2018; Zhao et al., 2021; Kamenova et al., 2023). The core concept of dynamic fixation lies in mitigating stress concentration by allowing limited segmental motion. However, some researchers are concerned that this design may alter screw stress distribution, increasing the risk of pedicle screw loosening (PSL) (Hekimoglu et al., 2024).

Currently, the differences between dynamic fixation and rigid fixation concerning PSL remain controversial. Our clinical findings suggest that while the incidence of PSL is relatively high following non-fusion fixation, many loosened screws gradually regain stability over extended follow-up. This phenomenon indicates that PSL after dynamic fixation may not signify permanent failure. Instead, screws may adapt to the biomechanical environment, facilitating re-establishment of tight contact between the screw threads and bone interface.

We hypothesize that the biomechanical differences between dynamic and rigid fixation may be critical factors contributing to this phenomenon. Therefore, investigating the biomechanical differences, particularly in screw stability and stress distribution, is essential.

This study employs FE analysis to simulate the biomechanical effects of these two fixation methods on the lumbar spine. It focuses on changes in stress transmission at the screw-bone interface, aiming to reveal the differences in PSL and stress distribution between dynamic and rigid fixation. These findings will provide scientific evidence to guide the clinical selection of appropriate surgical strategies.

## 2 Materials and methods

### 2.1 Development of an FE model of the lumbar-sacral spine

A three-dimensional FE model of the lumbar spine (L3 to sacrum S1) was developed based on CT scan data from a healthy adult male, with a slice thickness of 0.5 mm (Informed consent has been obtained from the volunteer). CT Imaging: Imaging was conducted using a Siemens dual-source CT scanner. The scanning parameters included a tube voltage of 120 kV and a tube current of 355 mA, with a slice thickness of 1 mm. CT images were processed into three-dimensional geometric models using Mimics 21.0 software, and surface reconstruction and smoothing were performed with Geomagic Studio software to create an anatomically accurate spinal model. The model was preprocessed for meshing using SolidWorks 2017 software. The cortical bone and trabecular bone were discretized using tetrahedral elements and assigned specific material properties. The final mesh was then generated using Ansys Workbench. The model included cortical bone, cancellous bone, posterior structures, intervertebral discs, and seven ligament types: anterior longitudinal ligament, posterior longitudinal ligament, ligamentum flavum, interspinous ligament, supraspinous ligament, intertransverse ligament, and capsular ligaments. All ligaments were modeled as tension-only spring elements.

**FIGURE 1:**
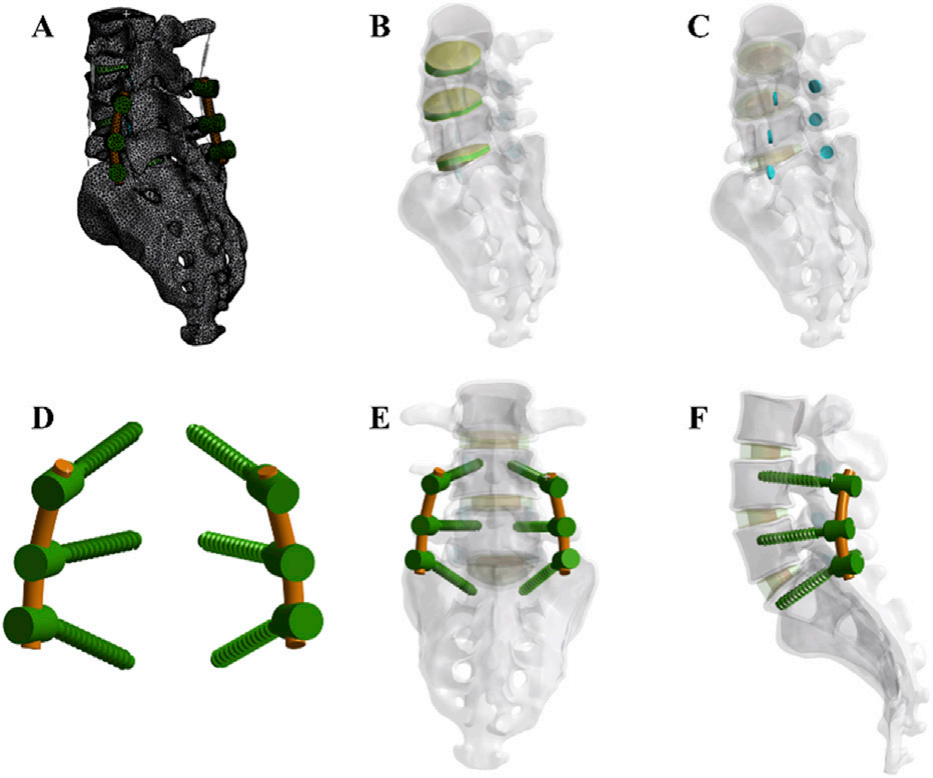
Development of the finite element (FE) model **(A)** The complete model after meshing **(B)** Integration of intervertebral discs within the overall model **(C)** Arrangement of facet cartilage within the complete model. **(D,E, F)** Assembly relationships of pedicle screws, rods, and the full model. Note: An axial compressive force of 300 N and a torque of 10,000 N mm were applied (flexion, extension, lateral bending, and rotation).

Material properties were assigned based on previously published studies (Ambati et al., 2015; Chen and Chang, 2021; Fan et al., 2021; Guo and Fan, 2018; Li et al., 2023; Li et al., 2024a; Yu Q. et al., 2024; Zhang et al., 2022), as summarized in Table 1. The intervertebral disc was modeled using an isotropic material model. Facet joint surfaces were modeled using a frictional contact interface with a coefficient of friction set at 0.1. The final lumbar-sacral spine model consisted of the L3-L5 vertebrae, sacrum, coccyx, and three intervertebral discs (Figure 1). The vertebral bodies of the lumbar, sacral, and coccygeal segments are composed of cortical bone and trabecular bone parts. The intervertebral discs are made up of the upper and lower endplates, the annulus fibrosus, and the nucleus pulposus. The entire lumbar-sacral spine model includes 4 cortical bone parts, 4 trabecular bone parts, 3 upper endplate parts, 3 lower endplate parts, 3 annulus fibrosus parts, 3 nucleus pulposus parts, 6 facet joint cartilage parts, 3 anterior longitudinal ligament parts, 3 posterior longitudinal ligament parts, 3 ligamentum flavum parts, 3 interspinous ligament parts, 3 supraspinous ligament parts, 6 intertransverse ligament parts, and 6 joint capsule ligament parts.

**TABLE 1:** Material properties of finite element models.

Material	Young’s modulus (MPa)	Poisson’s ratio	Cross-section area (mm^2^)
Cortical bone	12,000	0.3	—
Cancellous bone	100	0.2	—
Cartilage	10	0.4	—
Annuli fibrosi	4.2	0.45	—
Nuclei pulposi	1	0.49	—
Endplates	1,000	0.40	—
Pedicle screw (Titanium)	110,000	0.30	—
Rod (Titanium)	110,000	0.30	—
Rod (PEEK)	3,600	0.35	—
Anterior longitudinal	7.8 (<12.0%) 20.0 (>12.0%)	—	63.7
Posterior longitudinal	10.0 (<11.0%) 20.0 (>11.0%)	—	20
Ligamentum flavum	15.0 (<6.2%) 19.5 (>6.2%)	—	40
Capsular	7.5 (<25.0%) 32.9 (>25.0%)	—	30
Interspinous	10.0 (<14.0%) 11.6 (>14.0%)	—	40
Supraspinous	8.0 (<20.0%) 15 (>20.0%)	—	30
Transverse ligament	59	—	1.8

### 2.2 Development of FE models of the implanted lumbar-sacral spine

**FIGURE 2:**
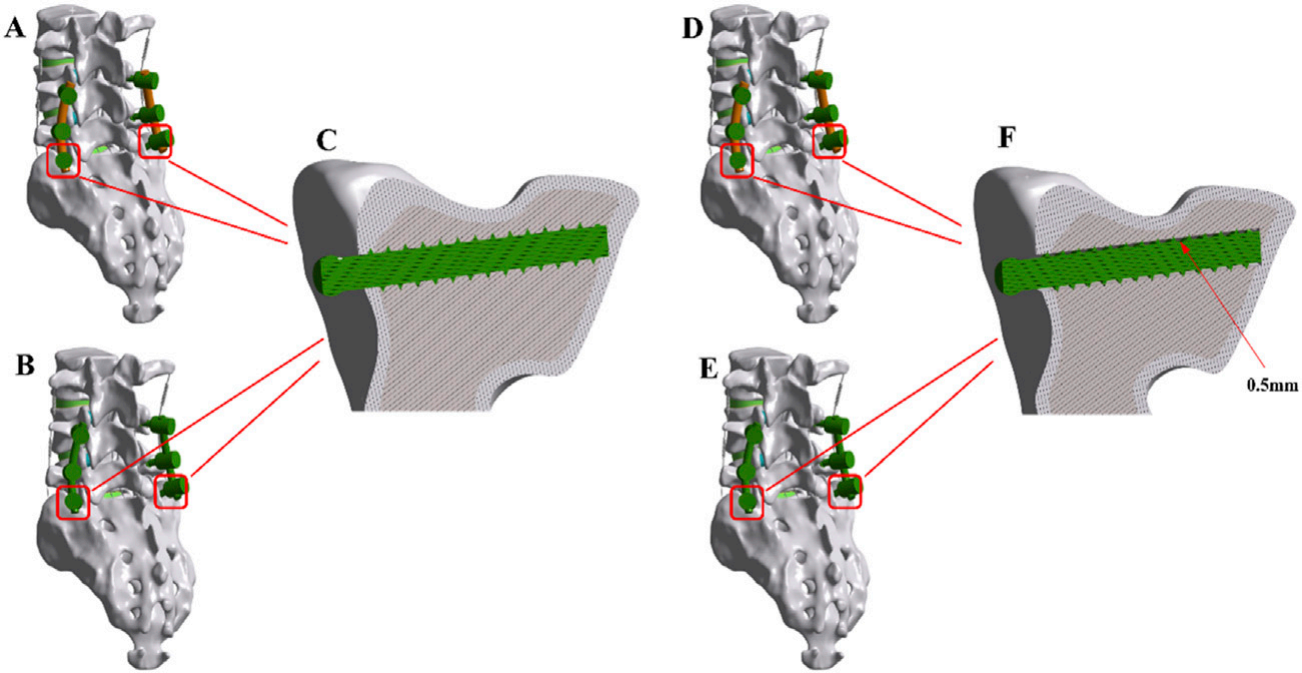
The finite element (FE) models of the implanted lumbar-sacral spine constructed for the present study. **(A)** PEEK rod dynamic fixation model without pedicle screw loosening. **(B)** Titanium rod rigid fixation model without pedicle screw loosening. **(C)** Model with tightly locked screws and sacrum. **(D)** PEEK rod dynamic fixation model with pedicle screw loosening. **(E)** Titanium rod rigid fixation model with pedicle screw loosening. **(F)** Model with a 0.5 mm gap between screws and sacrum.

Based on the baseline lumbar-sacral model, common spinal internal fixation procedures were simulated by implanting a pedicle screw-rod system. The study employed dual-segment fixation of the L4-S1 segment using the screw-rod system. PSL was modeled by introducing a 0.5 mm gap between bilateral S1 screws and the vertebral body. Titanium alloy pedicle screws were 6.5 mm in diameter, while both PEEK rods and titanium rods were 5.5 mm in diameter. All implants were meshed using three-dimensional solid elements. A bonded contact was assumed between screws and vertebrae, implying a tight, non-slipping interface. As shown in Figure 2.

FE models were developed for different surgical scenarios, including a PEEK rod dynamic fixation model (PEEK model), a PEEK rod dynamic fixation PSL model (PEEK-PSL model), a titanium rod rigid fixation model (titanium model), and a titanium rod rigid fixation PSL model (titanium-PSL model). These models simulated the biomechanical impacts of different fixation strategies on the lumbar-sacral spine.

### 2.3 FE analyses and validation

FE analysis was conducted using Ansys Workbench 2023 to simulate stress distribution and displacement responses under various loading conditions. An axial compressive force of 300 N was applied to the L3 vertebral body to simulate an upright posture. On this basis, a torque of 10,000 N mm was added to simulate four physiological motions: flexion, extension, lateral bending (left and right), and rotation (left and right). The boundary condition was set as complete fixation of the sacrum (S1).

The analysis outcomes included the range of motion (ROM) of the lumbar-sacral spine under different loads, stress distribution in the vertebrae and intervertebral discs, forces on the implants (screws and rods), and forces on the facet joints. Post-processing of the results was performed in Ansys Workbench 2023 to generate stress and displacement contour maps. The primary focus was on the stress variations in the implants post-surgery and the impact of different motion modes on the lumbar-sacral spine. By comparing the stress distribution between the baseline and implanted models, the study evaluated the biomechanical effects of the internal fixation system on the spine. Stress concentration areas were identified and analyzed for their potential clinical implications, such as pedicle screw loosening or fracture.

### 2.4 Data analyses

All statistical analyses were performed using SPSS 26.0 software. Hypothesis testing was conducted using two-tailed tests, with the significance level set at *P* < 0.05.

## 3 Results

### 3.1 Convergence test and validation

The difference in maximum displacement, maximum cage stress, and maximum contact pressure between 3,017,126 and 1,030,2686 nodes was 0.22%, 0.24%, and 0.31%, respectively. The results indicate that the mesh density of the FE model was sufficiently fine for computation. The number of nodes and elements in the finite element model are shown in Table 2, with all elements being of type Solid186.

**TABLE 2:** Number of nodes and elements in the FE model.

	PEEK	Titanium	PEEK-PSL	Titanium-PSL
Number of elements	3052927	3035506	3042123	3046305
Number of nodes	5347462	5310318	5321286	5356492

**FIGURE 3:**
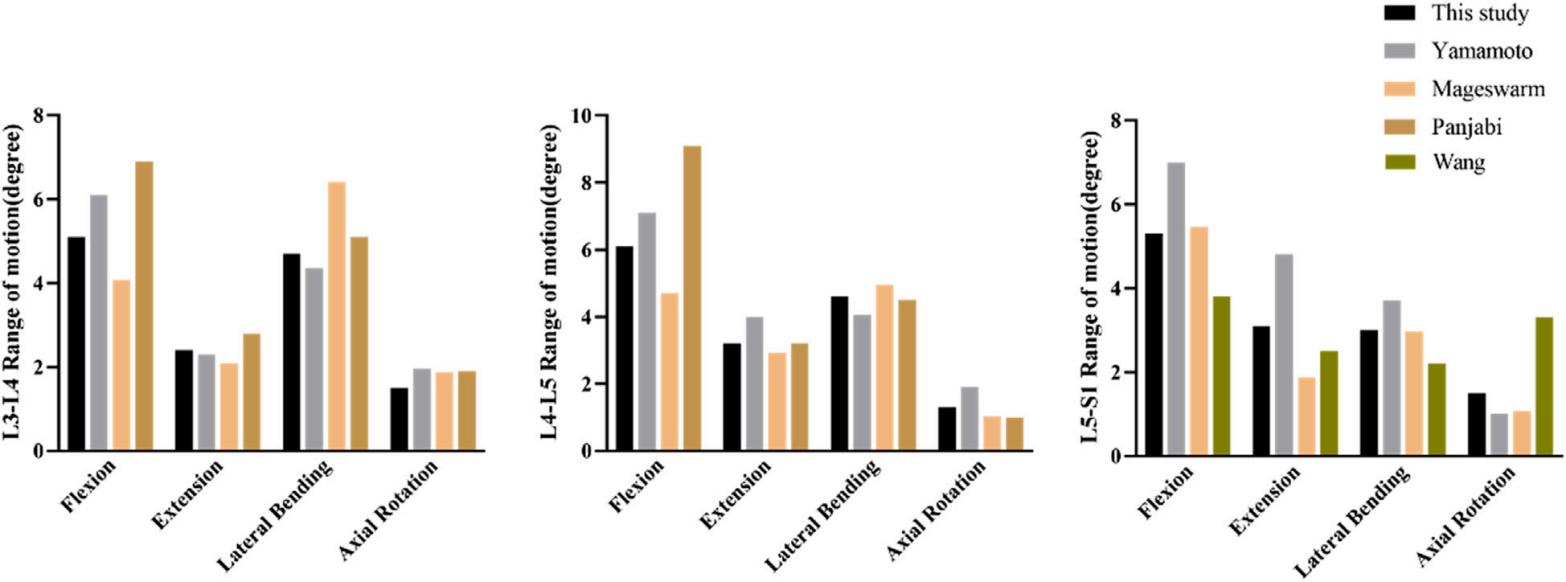
Comparison of the ROM in intact model with other ROMs in previous studies.

To validate the L3-S1 lumbar finite element model, the ROM of the model was calculated and compared with the ROM reported in previous *in vitro* studies and finite element analyses under similar loading conditions (Yamamoto et al., 1989; Panjabi et al., 1994; Mageswaran et al., 2012; Wang et al., 2013). As shown in Figure 3, there was a good agreement between the experimental and computational results. The ROM obtained in this study closely matched the findings of other studies, with errors within an acceptable range, demonstrating the high reliability of the model.

### 3.2 Stress on adjacent intervertebral discs and facet joints in fixed segments

**FIGURE 4:**
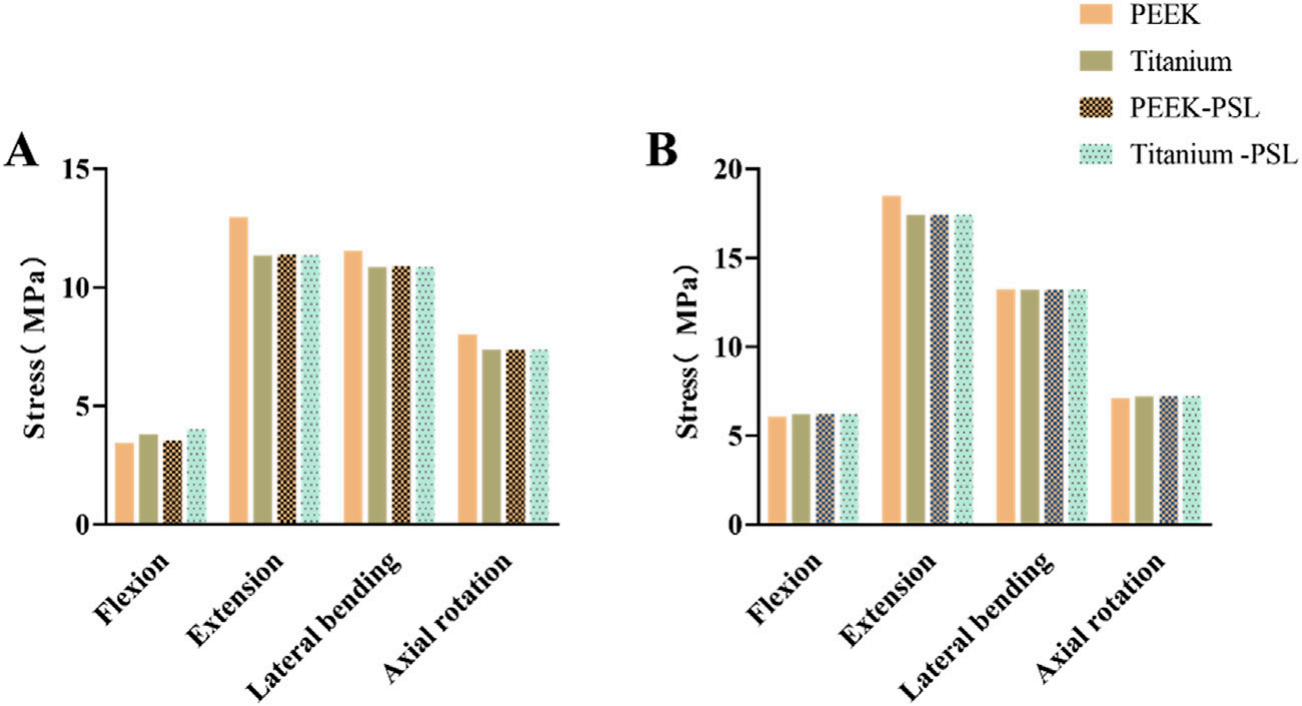
Average von Mises stress for the L3-L4 intervertebral disc and facet joints under four physiological conditions: **(A)** Average von Mises stress in the L3-L4 intervertebral disc for the four models; **(B)** Average von Mises stress in the facet joints for the four models.

**FIGURE 5:**
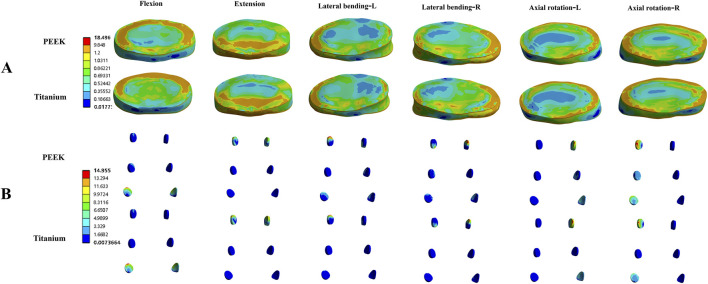
Von Mises stress (MPa) contour maps for the L3-L4 intervertebral disc **(A)** and facet joints **(B)** in the PEEK rod model and titanium rod model under four physiological conditions.

Figure 4 shows the average stress in the L3-L4 intervertebral disc and facet joints for the four models. Under all four physiological conditions, the average stress in the L3-L4 intervertebral disc and facet joints for the PEEK model was similar to that in the titanium model (stress distribution contour maps are shown in Figure 5). In the PEEK-PSL model and titanium-PSL model, the average stress in the L3-L4 intervertebral disc and facet joints also showed minimal differences, with no significant changes compared to the corresponding PEEK or titanium models.

### 3.3 Stress on the vertebral bodies and nucleus pulposus in fixed segments

**FIGURE 6:**
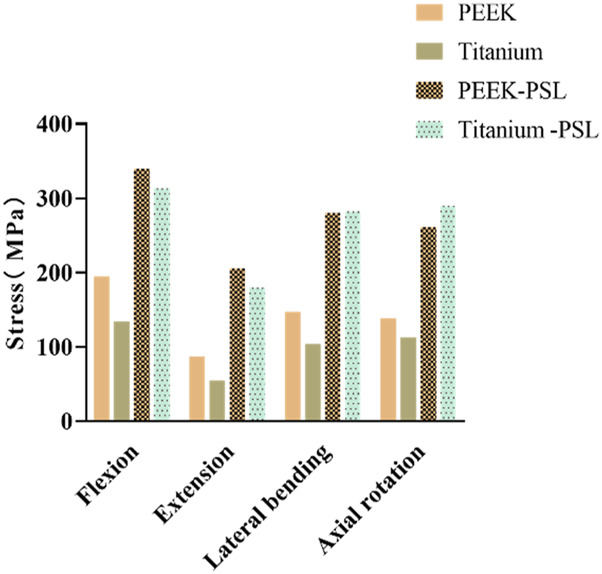
Average von Mises stress on the vertebral body in the four models under four physiological conditions.

Figure 6 shows the average stress on the vertebral body in the four models. Under all four physiological conditions, the average stress on the vertebral body in the PEEK model is significantly higher than that in the titanium model. In contrast, the average stress on the vertebral body is similar between the PEEK-PSL model and the titanium-PSL model.

**FIGURE 7:**
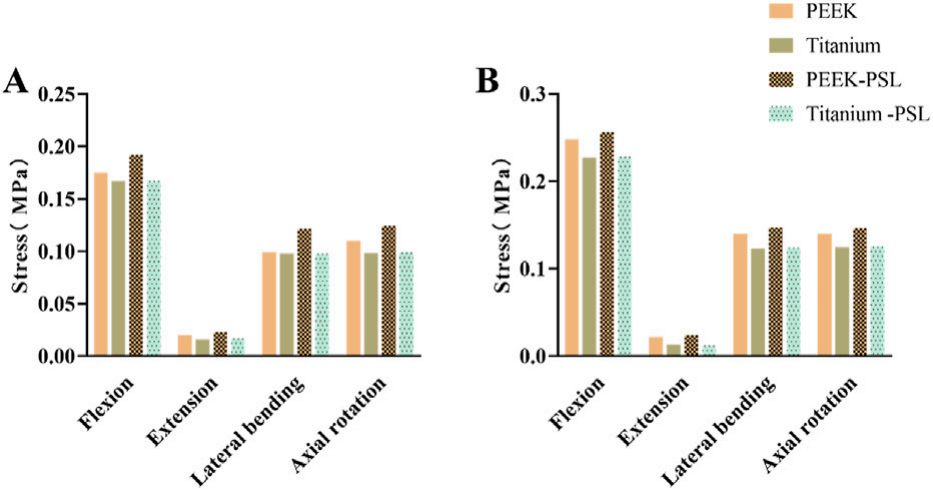
Average von Mises stress on **(A)** the nucleus pulposus of L4-L5 and **(B)** the nucleus pulposus of L5-S1 in the four models under four physiological conditions.

Figure 7 shows the average stress on the nucleus pulposus at the L4-5 and L5-S1 levels in the four models. Under all four physiological conditions, the average stress on the nucleus pulposus at both L4-5 and L5-S1 is very small in all four models. The PEEK model and titanium model exhibit almost identical stress values. Furthermore, after screw loosening, there is no noticeable change in the stress on the nucleus pulposus between the two models.

### 3.4 Stress on screws and rods

**FIGURE 8:**
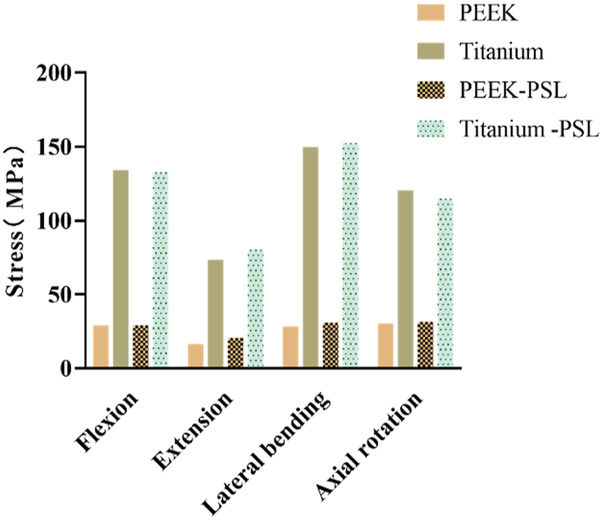
Average von Mises stress on the rods in the four models under four physiological conditions.

**FIGURE 9:**
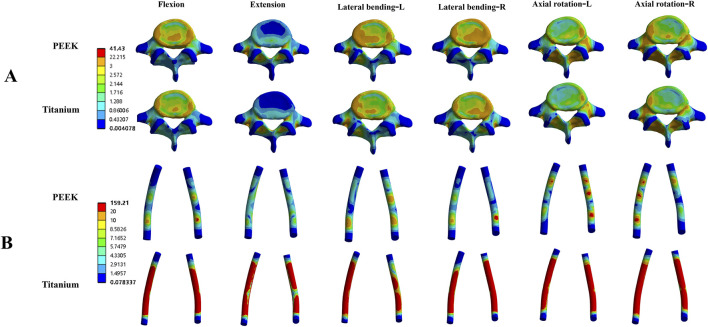
Von Mises stress (MPa) distribution cloud maps for the L5 vertebral body **(A)** and rods **(B)** in the PEEK and titanium models under four physiological conditions.

In the four models, the average stress distribution on the rods is shown in Figure 8. Under all four physiological conditions, the average stress on the rods in the titanium model was 3–4 times higher than that in the PEEK model. Similarly, in the titanium-PSL model, the average stress on the rods was significantly higher than that in the PEEK-PSL model. The stress distribution on the vertebrae and rods across the four models is illustrated in Figure 9.

**FIGURE 10:**
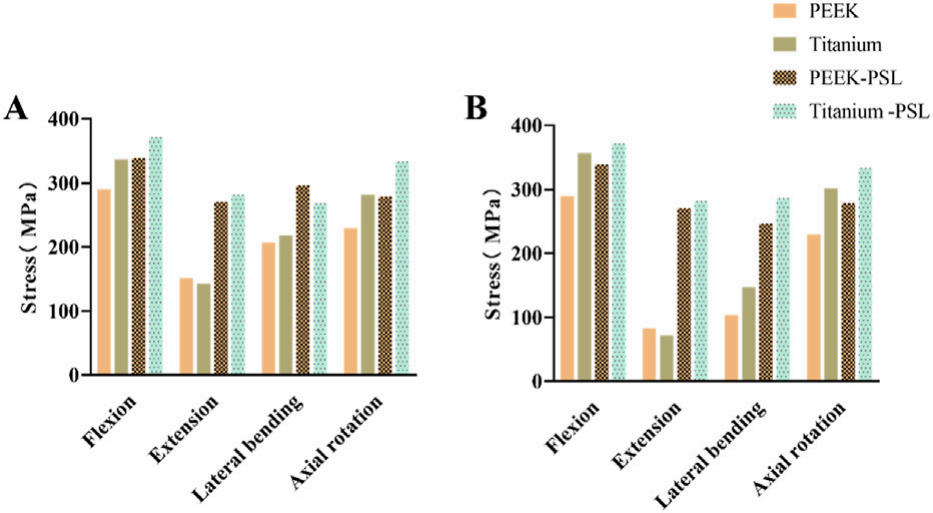
**(A)** Average von Mises stress of screws overall and **(B)** average von Mises stress of S1 loose screws in the four models under four physiological conditions.

**FIGURE 11:**
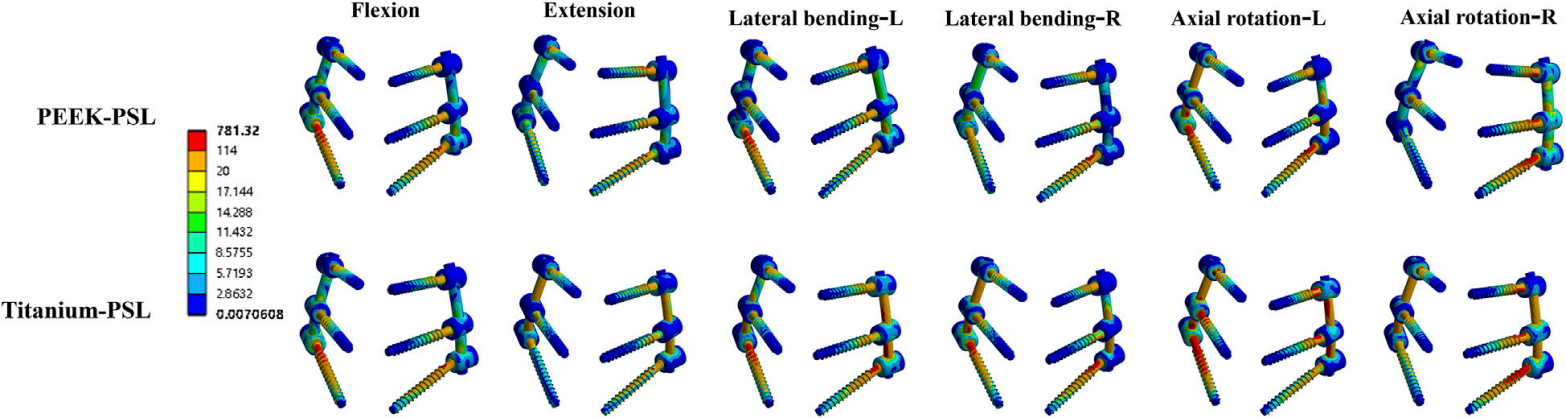
Von Mises stress (MPa) distribution of screws in the PEEK rod loose screw model and titanium rod loose screw model under four physiological conditions.

The average stress distribution on the screws overall and the loosened S1 screws in the four models is presented in Figure 10. For the screws overall, the average stress in the PEEK model was comparable to that in the titanium model under all four physiological conditions. After screw loosening, the average stress on the screws increased in both the PEEK rod and titanium rod models. For the S1 screws, under all four physiological conditions, the average stress in the PEEK-PSL model was slightly lower than that in the titanium-PSL model. However, in the PEEK-PSL model, the stress distribution on the S1 screws was more uniform, whereas in the titanium-PSL model, stress was concentrated at the rod-screw junction area (Figure 11).

## 4 Discussion

This study systematically investigated the biomechanical characteristics and the impact of screw loosening in lumbar spine fixation using PEEK and titanium rods through finite element analysis. The findings revealed differences in stress distribution and screw stability between fixation devices made of different materials. These differences provide valuable scientific insights and references for selecting fixation materials in various clinical scenarios, highlighting the potential for optimizing internal fixation devices for specific cases.

Spinal fusion surgery remains the gold standard for treating lumbar degenerative diseases due to its effective three-column fixation and maintenance of long-term lumbar stability (de Kunder et al., 2017; Kurra et al., 2018). However, ASD, a common complication following lumbar fusion, has mechanisms that remain unclear (Kim et al., 2016; Rudisill et al., 2024). Studies have reported an increased incidence of ASD after lumbar fusion, with researchers suggesting that fusion may elevate stress on adjacent segments, potentially accelerating their degeneration (Du et al., 2021; Rienmüller et al., 2019; Park et al., 2024). To prevent or delay ASD, the concept of “dynamic fixation” has emerged. Lumbar dynamic fixation systems aim to maintain stability at the fixed segment while preserving partial mobility, thereby reducing stress shielding. PEEK rods, with their high strength and elastic modulus similar to cortical bone, theoretically maintain lumbar stability while retaining some degree of motion. Some spine surgeons have attempted to use PEEK rods in non-fusion surgeries, achieving relatively satisfactory short-term clinical outcomes (Kurtz and Devine, 2007; Chou et al., 2015; Liu et al., 2018).

Some researchers argue that the occurrence of ASD is not necessarily associated with the type of spinal fixation method used (dynamic fixation or rigid fixation) (Donnally et al., 2020). The surgery itself may disrupt the biomechanical environment of adjacent segments, affect local blood supply, and alter the metabolism of cartilage and intervertebral disc cells, thereby accelerating degeneration. Pre-existing degenerative factors may worsen postoperatively, with mildly degenerated adjacent segments becoming more pronounced under altered stress conditions. Additionally, spinal degeneration is a progressive process, and adjacent segment degeneration may become more apparent over time following surgery. Various surgical factors, such as the number of fused segments, fusion level, and fixation method, may also influence the risk of adjacent segment degeneration.

The stress distribution results of this study indicate that different fixation methods do not cause significant differences in stress on the intervertebral disc and articular cartilage. This suggests that, for short-segment fixation, there is no significant difference in the incidence of ASD in adjacent segments between dynamic and rigid fixation. However, another study reported that for fixation extending to three or four segments, dynamic fixation significantly reduced stress on the intervertebral disc and articular cartilage of adjacent segments compared to rigid fixation (Li et al., 2024a).

Stress concentration after spinal fusion surgery may affect the long-term stability of implants, increase the risk of screw or rod breakage, and compromise surgical outcomes (Nowak, 2019; Li et al., 2022). To investigate the impact of different fixation methods on stress distribution, this study compared the stress distribution in titanium rod and PEEK rod fixation models after spinal fusion. The results showed that stress concentration was more pronounced in the high-rigidity titanium rod fixation model, with significantly higher stress observed in the rods compared to the PEEK model, potentially increasing the risk of screw or rod breakage. In contrast, the low elastic modulus and flexibility of PEEK rods resulted in a more uniform stress distribution in the dynamic fixation model, reducing stress concentration and offering potential advantages in extending the lifespan of the implants.

Stress shielding refers to the phenomenon where implanted devices bear the majority of the stress, leading to a reduction in the stress experienced by the surrounding bone tissue. This is a common side effect of high-rigidity material fixation (Kanayama et al., 2000; Ferri et al., 2023). Since normal bone metabolism and remodeling require adequate stress stimulation, excessive stress shielding can inhibit bone formation, resulting in osteoporosis around the implant site and increasing the risk of loosening and implant failure (Bulaqi et al., 2015). Titanium rods, due to their higher rigidity, are more likely to cause stress shielding. In contrast, the flexibility of PEEK material, with an elastic modulus closer to that of bone, theoretically reduces this effect. Nevertheless, screw loosening remains a common complication after surgery for both fixation methods, and there is no consensus on which fixation method results in a lower screw loosening rate (Zhang et al., 2024; Li Q. et al., 2024; Jiang et al., 2024).

Studies have reported differences in screw loosening rates between rigid fixation and dynamic fixation; however, few have explored the outcomes of screw loosening between these two fixation methods (Pham et al., 2016). Clinical observations by our team indicate that a certain proportion of screws loosen shortly after surgery in both PEEK rod dynamic fixation and titanium rod rigid fixation. Over time, most loosened screws in the PEEK rod group gradually regain stability, whereas only a small proportion of loosened screws stabilize in the titanium rod group. Previous studies have shown that appropriate mechanical stress stimulation can reduce the number and activity of osteoclasts, inhibit bone resorption, promote osteoblast differentiation and bone formation, suppress the differentiation of bone marrow mesenchymal stem cells into adipocytes, and prevent bone loss (Uzbekov et al., 2012; Li et al., 2020).

We hypothesize that this difference may arise because the elastic deformation of PEEK rods induces micro-movement of screws within the screw tract, facilitating bone-screw interface remodeling and accelerating the establishment of screw restabilization. Our FE analysis supports this possibility. Under simulated screw loosening conditions, stress distribution in the S1 screws of the PEEK-PSL model was more uniform, avoiding the stress concentration at the screw-rod junction observed in the titanium-PSL model. Additionally, we found that physiological activities such as flexion, lateral bending, and rotation increase local screw stress, while extension results in a more uniform stress distribution. These findings provide guidance for designing postoperative rehabilitation plans for patients.

Although PEEK rods have demonstrated potential advantages in dynamic fixation techniques, this study has certain limitations in model construction and simulation. For instance, the gap model used to simulate screw loosening cannot fully replicate the complex conditions of *in vivo* screw loosening, which is often accompanied by factors such as osteophyte formation and tissue healing in clinical scenarios. Moreover, the FE model did not account for individual patient differences or biological responses *in vivo*, which may influence stress transmission and screw stability to varying degrees.

Future studies could address these limitations by integrating *in vivo* experiments, animal models, and clinical follow-up in humans to further validate the reliability and applicability of the findings. Such efforts would provide more robust scientific evidence for the personalized selection of fixation devices, contributing to the precise treatment of lumbar degenerative diseases.

## 5 Conclusion

The PEEK rod fixation system reduces the risk of stress concentration through more uniform stress distribution, demonstrating superiority over the titanium rod fixation system in maintaining implant stability and reducing screw loosening rates. Titanium rods, on the other hand, offer advantages in fixation segments requiring higher rigidity and may be more suitable for patients needing stable structural support.

## Data Availability

The raw data supporting the conclusions of this article will be made available by the authors, without undue reservation.
